# Sex‐Specific Prevalence and Distribution of Electrocardiographic Variants in Healthy Vietnamese Freshmen

**DOI:** 10.1111/anec.70247

**Published:** 2026-10-22

**Authors:** Thang Nguyen Ngoc, Quyet Nguyen Minh, An Bui Thi Thu, Thang Vu Tien, Soan Hoang Thu, Cuong Duong Cao, Vy Nguyen Hoai, Cong Nguyen Thanh, Binh Tran Van, Anh Dinh The

**Affiliations:** ^1^ Emergency Department Phenikaa University Hospital Hanoi Vietnam; ^2^ College of Medicine Phenikaa University Hanoi Vietnam; ^3^ Department of Pediatrics and Neonatology Vinmec Central Park International Hospital Ho Chi Minh City Vietnam; ^4^ Outpatient Cardiology Department Vinmec International Hospital Hanoi Vietnam; ^5^ Department of Nephrology People's 115 Hospital Ho Chi Minh City Vietnam; ^6^ College of Medicine Taipei Medical University Taipei Taiwan; ^7^ Cardiac Surgical Intensive Department Cho Ray Hospital Ho Chi Minh City Vietnam

**Keywords:** early repolarization pattern, ECG variants, electrocardiography, freshmen, QT interval, right bundle branch block, sex differences, Vietnamese population

## Abstract

**Background:**

Data on ECG characteristics and qualitative ECG variants in healthy young Vietnamese adults remain limited. We evaluated sex‐specific ECG differences and factors independently associated with common ECG variants in first‐year Vietnamese university students.

**Methods:**

This cross‐sectional study included 6099 healthy students (3440 female [56.4%], 2659 male [43.6%]; mean age, 17.85 ± 0.65 years) undergoing standardized cardiovascular screening. Resting 12‐lead ECGs were analyzed for quantitative parameters and qualitative variants.

**Results:**

All four core ECG parameters differed by sex (all *p* < 0.001). Females had higher heart rates (84.64 vs. 81.55 bpm) and longer QTc intervals (423.54 vs. 406.46 ms), whereas males had longer PR intervals (141.99 vs. 138.99 ms) and wider QRS complexes (91.44 vs. 80.81 ms). Males had higher prevalences of sinus arrhythmia (22.7% vs. 19.2%), early repolarization pattern (6.1% vs. 0.1%), right bundle branch block (4.4% vs. 0.3%), and sinus bradycardia (4.5% vs. 1.3%; all *p* < 0.001). Male sex was independently associated with early repolarization pattern (aOR, 37.7; 95% CI, 17.2–107) and right bundle branch block (aOR, 13.1; 95% CI, 7.55–25.2; both *p* < 0.001), but not sinus arrhythmia (aOR, 1.01; 95% CI, 0.84–1.21; *p* = 0.896).

**Conclusions:**

ECG parameters and several qualitative variants differed by sex. These findings provide descriptive, population‐specific data on ECG characteristics and common qualitative variants in healthy young Vietnamese university students.

## Graphical abstract

This large‐scale study establishes sex‐specific ECG reference values in healthy young Vietnamese adults, identifying marked male predominance in early repolarization and bundle branch block, while sinus arrhythmia was primarily associated with heart rate and age. These findings support population‐ and sex‐specific ECG interpretation in Southeast Asian populations.

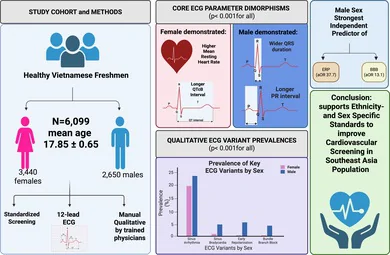

## 1 Introduction

The 12‐lead electrocardiogram (ECG) remains one of the most widely utilized non‐invasive tools for cardiovascular screening, arrhythmia detection, and risk stratification. Interpretation of ECG findings relies heavily on established normative reference ranges; however, accumulating evidence demonstrates that ECG parameters vary substantially according to age (Tseng et al. 2020; Surawicz and Parikh 2002), sex (Ngoc et al. 2025), ethnicity (Walsh et al. 2019), and autonomic state (Mühl et al. 2023). As a result, the use of generalized reference standards derived predominantly from Western populations may lead to diagnostic misclassification when applied to underrepresented ethnic groups.

Sex‐related differences in cardiac electrophysiology are particularly well established (Ngoc et al. 2025; Mühl et al. 2023; Moss 2010; Andrade et al. 2018; Dewhurst et al. 2014; Braschi et al. 2017). Females generally exhibit higher resting heart rates (HR) and longer corrected QT intervals, whereas males demonstrate longer PR intervals, wider QRS complexes, and higher prevalences of conduction abnormalities and early repolarization patterns (ERP) (Ngoc et al. 2025; Palhares et al. 2017; Ahmadi et al. 2023). These differences are believed to reflect complex interactions among cardiac size, autonomic regulation, ion channel expression, and sex hormone influences on myocardial conduction and repolarization (Dogan et al. 2016; Bett 2016; Bidoggia et al. 2000). Importantly, many ECG variants considered benign in young adults—such as sinus arrhythmia (SA) (Corîci et al. 2018), early repolarization pattern (Bidoggia et al. 2000), and incomplete right bundle branch block (IRBBB) (Alventosa‐Zaidin et al. 2019) —show marked variation by sex and age. Characterizing the prevalence and distribution of these findings is therefore important for distinguishing common ECG phenotypes from potentially clinically significant abnormalities in young healthy populations. Despite extensive ECG research in European, North American, and East Asian populations, data describing the prevalence of qualitative ECG variants in healthy young Vietnamese freshmen remain limited. This study extends our prior work establishing sex‐specific baseline ECG profiles in 5127 healthy Vietnamese university students by including an additional 1046 participants and characterizing the prevalence of qualitative ECG variants and their independent correlates. These findings provide a more detailed descriptive framework for understanding ECG characteristics in this population and may offer useful comparative data for Vietnamese and neighboring Southeast Asian populations.

## 2 Method

### 2.1 Study Design and Participant

#### 2.1.1 Study Setting and Design

This was a cross‐sectional observational study conducted as part of the mandatory annual pre‐semester health screening programme at Phenikaa University, Hanoi, Vietnam, in collaboration with Phenikaa University Hospital.

#### 2.1.2 Participant Selection

To ensure a homogeneous, healthy reference cohort, participants were required to meet all of the following pre‐specified criteria: Vietnamese ethnicity; age 17–29 years; asymptomatic at evaluation with a documented normal physical examination; no personal or family history of cardiovascular disease, inherited arrhythmia syndromes, metabolic disease, or genetic disorders; BMI 18–25 kg/m^2^; normotensive at screening; no current use of medications or hormonal agents known to influence cardiac electrophysiology. Female participants were additionally excluded if currently menstruating or pregnant, as confirmed by self‐report, to minimize transient hormonal influences on repolarisation. Of 6173 ECG recordings obtained, 74 were excluded for technical artifacts or inadequate signal quality, yielding a final analytic cohort of 6099 participants.

### 2.2 ECG Measurement and Data Collection

Resting 12‐lead ECGs were recorded supine using the MAC 5 Resting ECG System (GE Healthcare, USA; 500 Hz sampling rate) by trained nursing staff, with standardized electrode placement and coded per the Minnesota Code classification system. Core quantitative parameters—HR, PR interval, QRS duration, and QTcB—were automatically extracted via the GE Marquette 12SL algorithm. Qualitative ECG variants were identified by independent manual review performed by three trained physicians, each blinded to participant sex and to the classifications of the other reviewers. Discrepancies among the three independent reviews were adjudicated by a fourth, independent cardiologist, whose classification was used as the final determination for analysis. All measurements were standardized and rounded to the nearest 0.5 mm (0.05 mV) for inter‐rater consistency.

### 2.3 Definition of ECG Abnormalities

#### 2.3.1 Sinus Arrhythmia

SA was defined as a sinus rhythm with variation in the P–P interval exceeding 120 ms (or > 10%) between the longest and shortest cycles, typically associated with the respiratory cycle in young individuals.

#### 2.3.2 Early Repolarization Pattern (ERP)

ERP was defined in accordance with current consensus criteria (Macfarlane et al. 2015). J‐point amplitude was measured relative to the PR segment (isoelectric baseline), rather than the TP segment, given the potential instability of the TP segment at higher HR. The J‐point was defined as the point of first inflection or change in slope following the QRS complex, marking the transition from the QRS to the ST segment. ERP was defined as J‐point elevation ≥ 0.1 mV in at least two anatomically contiguous leads, inferior (II, III, aVF) or lateral (I, aVL, V4–V6), manifesting as either slurring (a smooth transition from the QRS to the ST segment without a distinct notch) or notching (a positive deflection interrupting the terminal QRS, peaking ≥ 0.1 mV above baseline). Leads V1–V3 were excluded from this analysis to avoid overlap with right precordial ST‐elevation patterns, including the Brugada pattern.

#### 2.3.3 Bundle Branch Blocks (BBB)

Conduction delays involving the right bundle branch were categorized as follows:

- Complete Right Bundle Branch Block (CRBBB): QRS duration ≥ 120 ms with an rsr', rsR', or rSR' pattern in leads V_1_ or V_2_, and a wide, slurred S wave in leads I and V_6_ (Minnesota Code 7‐2‐1).
- Incomplete Right Bundle Branch Block (IRBBB): The same morphological criteria as CRBBB, but with a QRS duration of 100–119 ms (Minnesota Code 7–3).

#### 2.3.4 Atrioventricular (AV) Blocks and Conduction Delays

- First‐Degree AV Block: PR interval > 200 ms with 1:1 AV conduction (Minnesota Code 6–1).
- Second‐Degree AV Block (Mobitz Type II): Intermittent non‐conduction of P waves to the ventricles without progressive PR prolongation, with a constant PR interval in conducted beats (Minnesota Code 6‐2‐2).
- Left Anterior Fascicular Block (LAFB): Mean QRS axis between −45° and −90°, a qR pattern in lead aVL, and an rS pattern in leads II, III, and aVF (Minnesota Code 7‐1‐1).

#### 2.3.5 Premature Atrial Complexes (PACs)

PACs were identified as premature P waves of morphology differing from the dominant sinus P wave typically followed by a narrow QRS complex. Their presence was recorded as positive if one or more occurred within the standard 10‐s recording window.

#### 2.3.6 Wolff‐Parkinson‐White (WPW) Pattern

The WPW pattern was defined by the classic triad of: (Tseng et al. 2020) short PR interval (< 120 ms), (Surawicz and Parikh 2002) a slurred initial QRS deflection (delta wave), and (Ngoc et al. 2025) prolonged QRS duration (> 110 ms in young adults) (Minnesota Code 6‐4‐1).

#### 2.3.7 Ventricular Premature Contractions (VPC) Subtypes

VPCs were identified by premature, wide QRS complexes (> 120 ms) with morphology differing from sinus‐conducted beats and no preceding P wave. VPCs were further categorized by frequency and pattern during the 10‐s recording:

- Single (Isolated) VPC: Exactly one VPC throughout the entire tracing.
- Occasional VPC: More than one VPC without a repetitive, patterned sequence.
- Ventricular Bigeminy: A repetitive pattern in which every sinus beat is followed by a VPC (Minnesota Code 8‐1‐2).
- Ventricular Trigeminy: A repetitive pattern in which every two sinus beats are followed by a VPC (Minnesota Code 8‐1‐2).

#### 2.3.8 Definition of Prolonged and Short QTc

QTc was calculated using both Bazett's (QTcB) and Fridericia's (QTcF) formulae because of the relatively high resting HR in this cohort and the tendency of Bazett's formula to overcorrect at higher HR. QTcB was retained as the primary method for consistency with established normative and clinical literature, while QTcF was used for sensitivity analysis.

For QTcB, prolonged QTc was defined as ≥ 480 ms in females and ≥ 470 ms in males, and short QTc as < 360 ms in females and < 350 ms in males, consistent with common screening thresholds. Sex‐specific QTcF thresholds were derived from the cohort's 1st and 99th percentiles: < 360 ms (female) and < 348 ms (male) for short QTcF, and > 441 ms (female) and > 427 ms (male) for prolonged QTcF. These cohort‐derived thresholds were used only to assess consistency of prevalence estimates between correction methods and are not proposed as screening or diagnostic criteria. QTcB values were automatically extracted using the GE Marquette 12SL algorithm, while QTcF was calculated automatically from the raw HR and RR interval values. All flagged measurements were manually verified, and participants meeting either QTcB or QTcF threshold underwent secondary review by the adjudicating cardiologist.

#### 2.3.9 Definition of Sinus Bradycardia

Sinus bradycardia was evaluated using two predefined thresholds: resting HR below 60 bpm for conventional sinus bradycardia and below 50 bpm for marked sinus bradycardia, with both thresholds analyzed separately to distinguish common physiological reductions in resting HR from more pronounced bradycardic patterns.

### 2.4 Statistical Analysis Methods

Continuous variables were assessed for distribution using histograms and Q‐Q plots and are presented as mean ± standard deviation (SD). Between‐sex comparisons were performed using the Student's *t*‐test or Wilcoxon rank‐sum test, as appropriate. Categorical variables are presented as frequencies and percentages. Prevalence estimates are reported with 95% confidence intervals (CIs) calculated using the Wilson score method, and between‐sex differences were assessed using Pearson's chi‐square or Fisher's exact test, as appropriate.

Multivariable logistic regression was used to evaluate independent associations between sex and ERP, RBBB, and SA. Age, sex, BMI, and resting HR were selected a priori based on established clinical literature and physiological relevance to cardiac electrophysiology and autonomic function. Adjusted odds ratios (aORs) with 95% CIs were reported. Given the sparse events and marked sex imbalance for some outcomes, particularly ERP, Firth‐penalized logistic regression was performed as a sensitivity analysis to assess the robustness of conventional maximum‐likelihood estimates. Model calibration was assessed using the Hosmer–Lemeshow goodness‐of‐fit test, and McFadden's pseudo‐*R* ^2^ was reported as a measure of model fit. Potential non‐linear associations with age were assessed by adding quadratic age terms, and sex‐by‐age interaction terms were tested to assess potential effect modification. For ERP, interaction models were additionally examined for numerical instability and quasi‐complete separation because of the sparse number of events; where substantial instability was observed, the additive model was retained as the primary analysis.

For all analyses, a two‐sided *p* < 0.05 was defined as the threshold for statistical significance.

## 3 Results

### 3.1 Study Population and Baseline Characteristics

The final analytic cohort comprised 6099 participants: 3440 female (56.4%) and 2659 male (43.6%), with a median age of 18 years and an age range of 17–28 years. The sample is heavily concentrated in the late‐adolescent range: 17 years, 23.6% (*n* = 1442); 18 years, 70.8% (*n* = 4320); 19 years, 4.0% (*n* = 246); and only 1.5% (*n* = 91) aged 20 years or older. Female and male students were highly comparable in mean age (17.85 ± 0.62 vs. 17.85 ± 0.68 years; *p* = 0.916) and BMI distribution (*p* = 0.677) (Table 1), confirming a demographically homogeneous study population.

**TABLE 1:** Baseline characteristics and core ECG parameters by sex.

Characteristics^a^	Total population (*N* = 6099)^a^	Female (*N* = 3440)^a^	Male (*N* = 2659)^a^	*p* ^a^
Age (years), mean (SD)	17.85 (0.65)	17.85 (0.62)	17.85 (0.68)	0.916
Body mass index (kg/m^2^), mean (SD)	21.57 (2.07)	21.58 (2.09)	21.56 (2.04)	0.677
Heart rate (bpm), mean (SD)	83.29 (14.22)	84.64 (13.66)	81.55 (14.73)	< 0.001
PR interval (ms), mean (SD)	140.30 (17.85)	138.99 (17.60)	141.99 (18.03)	< 0.001
QRS duration (ms), mean (SD)	85.45 (10.20)	80.81 (8.16)	91.44 (9.43)	< 0.001
QTcB interval (ms), mean (SD)	416.09 (22.80)	423.54 (21.02)	406.46 (21.36)	< 0.001
QTcF interval (ms), mean (SD) by	394.75 (18.63)	400.69 (17.68)	387.06 (16.94)	< 0.001

*Note:* Values are mean (SD) for all continuous variables. *p*‐values for continuous variables were calculated using the two‐tailed independent samples Student's *t*‐test; BMI distribution was compared using the Pearson *χ* ^2^ test.

Abbreviations: BMI, body mass index; QTcB, Bazett‐corrected QT interval; QTcF, Fridericia‐corrected QT interval; SD, standard deviation.

^a^ Values are mean (SD). *p*‐values calculated via Student's *t*‐test.

### 3.2 Core ECG Parameters by Sex

Significant sex‐related differences were observed across all primary ECG parameters (Table 1 and Figure 1). Female students had a significantly higher mean resting HR than males (84.64 ± 13.66 vs. 81.55 ± 14.73 bpm; *p* < 0.001). Males exhibited longer atrial and ventricular conduction times: the mean PR interval was significantly longer in males (141.99 ± 18.03 vs. 138.99 ± 17.60 ms; *p* < 0.001), and the mean QRS duration was notably wider in the male cohort (91.44 ± 9.43 vs. 80.81 ± 8.16 ms; *p* < 0.001). Conversely, the QTcB interval was significantly longer in females (423.54 ± 21.02 vs. 406.46 ± 21.36 ms; *p* < 0.001). These findings demonstrate sex‐related differences in cardiac electrophysiology within a healthy cohort of first‐year Vietnamese university students.

**FIGURE 1:**
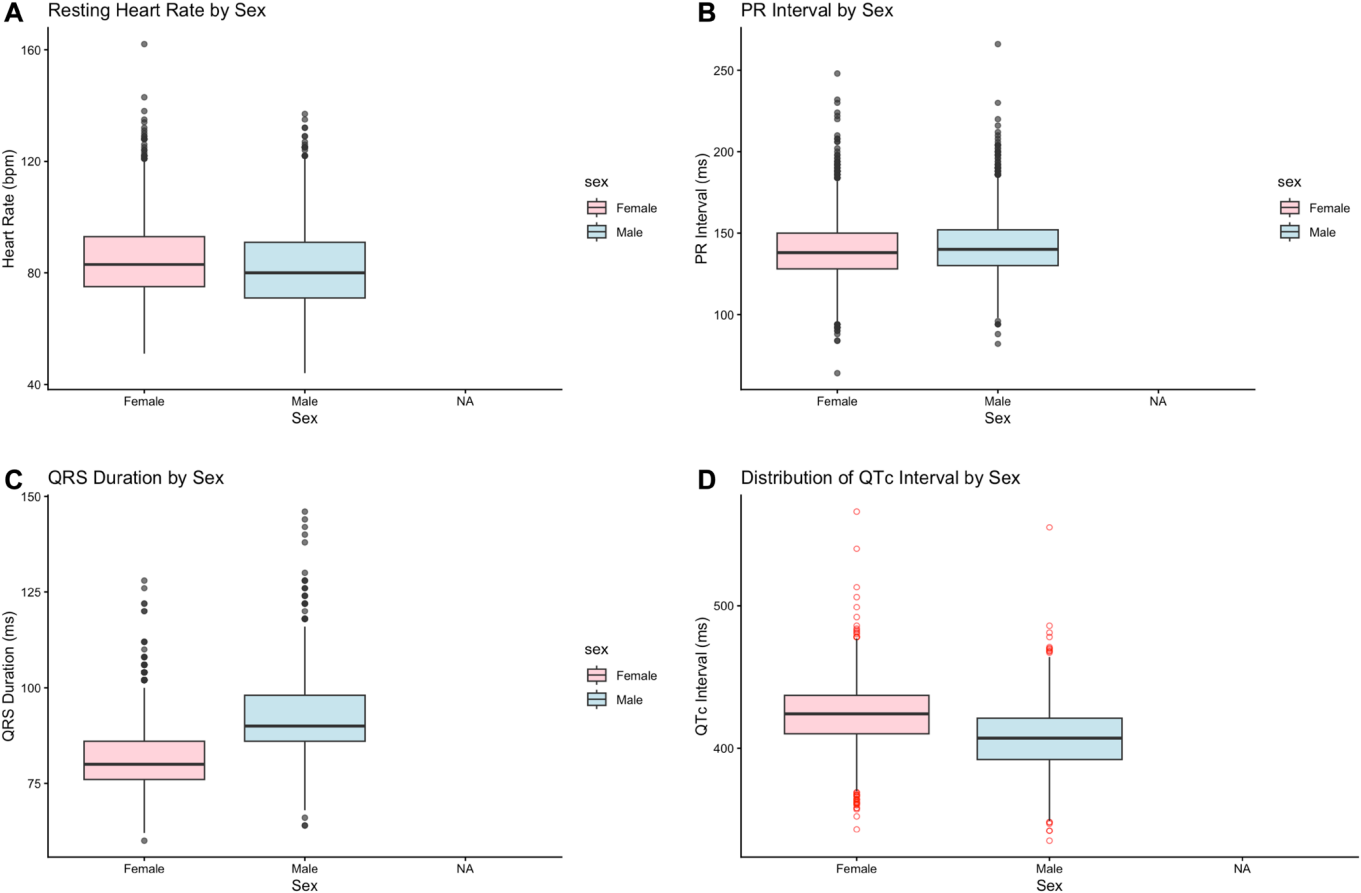
Displays the frequency distributions of the four core quantitative ECG parameters—resting heart rate (bpm), PR interval (ms), QRS duration (ms), and Bazett‐corrected QT interval (QTcB, ms)—separately for female (red) and male (blue) participants. For each parameter, the distributions are illustrated as overlapping histograms or kernel density plots to facilitate visual comparison between sexes. Vertical dashed lines indicate the sex‐specific mean values. All four parameters showed statistically significant sex‐based differences (*p* < 0.001 for each). Females demonstrated higher mean heart rates and longer QTcB intervals, while males demonstrated longer PR intervals and wider QRS complexes, consistent with established cardiac sexual differences.

### 3.3 Prevalence of ECG Variants by Sex

The prevalence of all identified ECG variants, stratified by sex, is summarized in Table 2.

**TABLE 2:** Prevalence of ECG variants by sex with 95% confidence intervals.

ECG finding	Total population (*N* = 6099)^a^	95% CI	Female (*N* = 3440)^a^	95% CI	Male (*N* = 2659)^a^	95% CI	*p* ^b^
Sinus arrhythmia	1264 (21%)	20%, 22%	660 (19%)	18%, 21%	604 (23%)	21%, 24%	< 0.001
Early repolarization pattern	166 (2.7%)	2.3%, 3.2%	5 (0.1%)	0.05%, 0.36%	161 (6.1%)	5.2%, 7.0%	< 0.001
Sinus bradycardia (< 60 bpm)	163 (2.7%)	2.3%, 3.1%	43 (1.3%)	0.92%, 1.7%	120 (4.5%)	3.8%, 5.4%	< 0.001
Sinus bradycardia (< 50 bpm)	3 (< 0.1%)	0.01%, 0.16%	0 (0%)	0.00%, 0.14%	3 (0.1%)	0.03%, 0.36%	0.083
Prolonged QTcB interval	19 (0.3%)	0.19%, 0.50%	12 (0.3%)	0.19%, 0.63%	7 (0.3%)	0.12%, 0.57%	0.6
Short QTcB interval	12 (0.2%)	0.11%, 0.35%	5 (0.1%)	0.05%, 0.36%	7 (0.3%)	0.12%, 0.57%	0.4
Prolonged QTcF interval (> 427 ms M/> 441 ms F)	65 (1.1%)	0.82%, 1.36%	38 (1.1%)	0.78%, 1.51%	27 (1.0%)	0.67%, 1.47%	0.802
Short QTcF interval (< 348 ms M/< 360 ms F)	64 (1.0%)	0.81%, 1.34%	35 (1.0%)	0.71%, 1.41%	29 (1.1%)	0.73%, 1.56%	0.801
Any ventricular premature contractions	21 (0.3%)	0.22%, 0.54%	11 (0.3%)	0.17%, 0.59%	10 (0.4%)	0.19%, 0.72%	0.8
Isolated (single) VPC	16 (0.3%)	0.16%, 0.44%	7 (0.2%)	0.09%, 0.44%	9 (0.3%)	0.17%, 0.67%	0.3
Occasional VPC	2 (< 0.1%)	0.01%, 0.13%	2 (< 0.1%)	0.01%, 0.23%	0 (0%)	0.00%, 0.18%	0.5
Repetitive patterns (bigeminy/trigeminy)	3 (< 0.1%)	0.01%, 0.16%	2 (< 0.1%)	0.01%, 0.23%	1 (< 0.1%)	0.00%, 0.24%	> 0.9
Right bundle branch block	130 (2.1%)	1.8%, 2.5%	12 (0.3%)	0.19%, 0.63%	118 (4.4%)	3.7%, 5.3%	< 0.001
AV block/conduction delay	20 (0.3%)	0.21%, 0.52%	8 (0.2%)	0.11%, 0.48%	12 (0.5%)	0.24%, 0.81%	0.2
Premature supraventricular complexes	21 (0.3%)	0.22%, 0.54%	15 (0.4%)	0.25%, 0.74%	6 (0.2%)	0.09%, 0.52%	0.2
Wolff‐Parkinson‐white pattern	6 (< 0.1%)	0.04%, 0.23%	1 (< 0.1%)	0.00%, 0.19%	5 (0.2%)	0.07%, 0.47%	0.092
Atrial fibrillation	0 (0%)	0.00%, 0.08%	0 (0%)	0.00%, 0.14%	0 (0%)	0.00%, 0.18%	> 0.9

*Note:* Values are *n* (%). 95% confidence intervals were calculated using the Wilson score method. *p*‐values were calculated using Fisher's exact test or Pearson's *χ* ^2^ test when appropriate. Prolonged QTcB defined as ≥ 480 ms in females and ≥ 470 ms in males; Short QTcB defined as < 360 ms in females and < 350 ms in males. Prolonged QTcF Interval defined as > 441 ms in females and > 427 ms in males. Short QTcF is defined as < 360 ms in females and 348 ms in males.

Abbreviations: AV, atrioventricular; CI, confidence interval; QTcB, Bazett‐corrected QT interval; QTcF, Fridericia‐corrected QT interval; RBBB, right bundle branch block; VPC, ventricular premature contraction.

^a^ *n* (%).

^b^ Fisher's exact test or Pearson's *χ* ^2^ test.

#### 3.3.1 Sinus Arrhythmia

SA was the most prevalent ECG variant in the cohort, identified in 21% of all participants. It was significantly more common in males than in females (23% vs. 19%; *p* < 0.001) (Table 2 and Figure 2).

**FIGURE 2:**
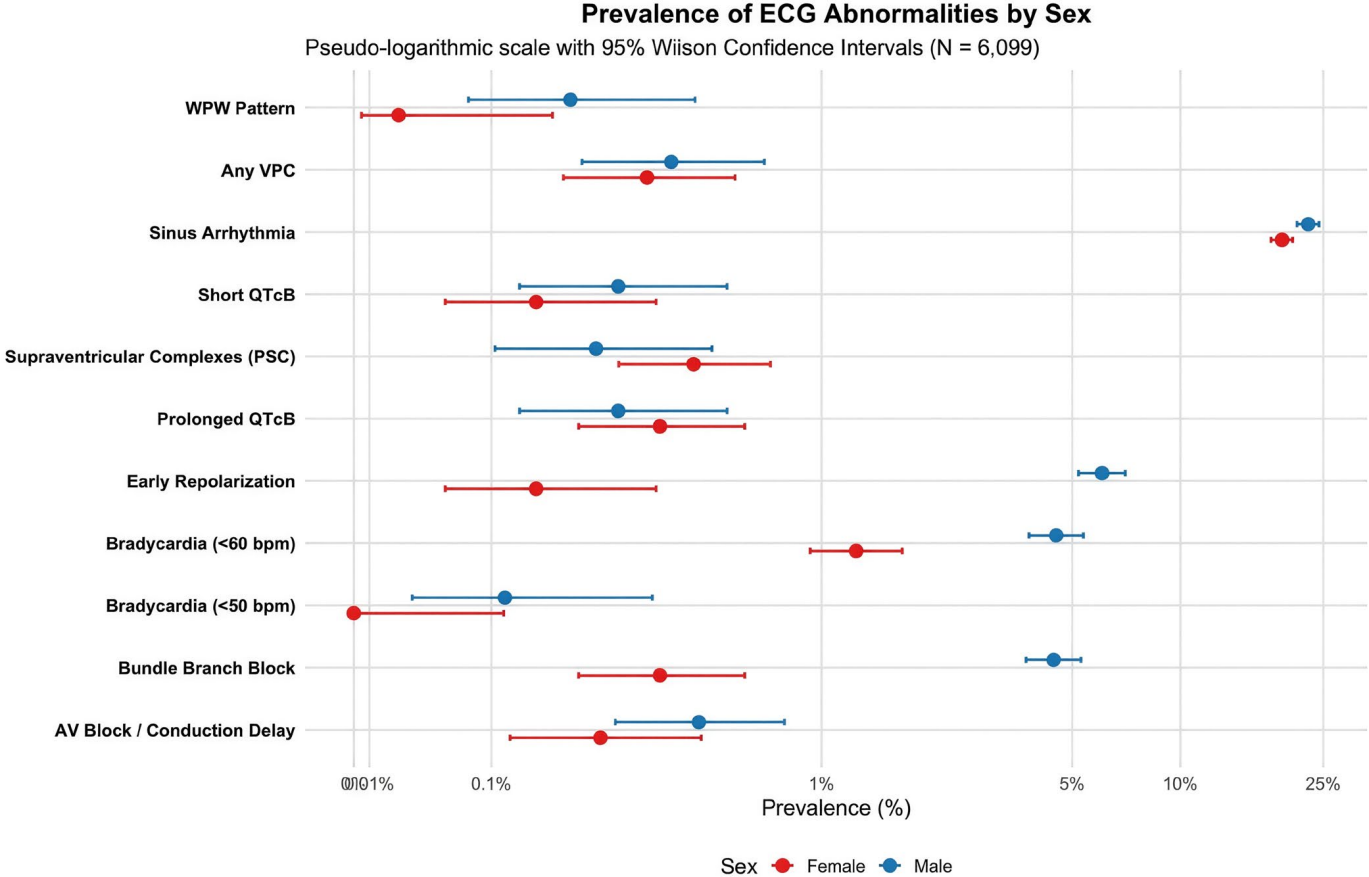
Prevalence of ECG abnormalities stratified by sex. Point estimates and 95% Wilson confidence intervals for 11 targeted ECG abnormalities evaluated in a cohort of 6099 individuals. Blue markers denote prevalence estimates for males, while red markers denote estimates for females. Data are plotted on a pseudo‐logarithmic horizontal scale to visualize both rare (< 0.1%) and common (> 10%) ECG patterns on a single axis.

#### 3.3.2 Early Repolarization Pattern

A striking sex‐based disparity was observed in the prevalence of the ERP. Males exhibited a substantially higher prevalence compared to females (6.1% vs. 0.1%; *p* < 0.001) (Table 2 and Figure 2), reflecting an approximately 40‐fold higher prevalence in the male cohort.

#### 3.3.3 Bundle Branch Block

A significant sex disparity was observed in the prevalence of intraventricular conduction delays. Any RBBB was identified in 4.4% of males compared to only 0.3% of females (*p* < 0.001). IRBBB was the predominant subtype, occurring in 4.2% of male students (*n* = 111) versus 0.3% of females (*n* = 10; *p* < 0.001). CRBBB was rare in both groups but remained significantly more prevalent in males (0.3%, *n* = 7) than in females (0.1%, *n* = 2; *p* = 0.047) (Table 2 and Figure 2). Notably, despite the overall male predominance, the ratio of incomplete to complete block did not differ significantly between sexes, with IRBBB accounting for more than 90% of all bundle branch abnormalities in both groups. No cases of left bundle branch block (LBBB) were detected.

#### 3.3.4 Ventricular Premature Contractions

VPCs were identified in only 0.3% (*n* = 21) of the total cohort and showed no significant sex difference in overall prevalence (males 0.4% vs. females 0.3%; *p* = 0.826). Among subtypes, isolated single VPCs were most common (0.3% in males, 0.2% in females; *p* = 0.324). Complex forms, including bigeminy and trigeminy, were exceedingly rare, each observed in fewer than 0.1% of either sex (*p* > 0.999) (Table 2 and Figure 2). The overall pattern of VPC subtypes did not differ significantly between sexes (simulated *p* = 0.495).

#### 3.3.5 Atrioventricular Blocks and Conduction Delays

AV conduction disturbances were identified in 0.3% (*n* = 20) of the study population, with no statistically significant sex difference in combined prevalence (males 0.5% vs. females 0.2%; *p* = 0.175). First‐degree AV block was the most common finding (0.2% of the total cohort; 0.2% in both sexes; *p* > 0.999). LAFB was present in 6 students (0.1%); although the prevalence appeared higher in males (0.2%, *n* = 5) than in females (< 0.1%, *n* = 1), this difference did not reach statistical significance (*p* = 0.092) (Table 2 and Figure 2). A single case of second‐degree AV block (Mobitz Type II) was identified in a male student. No cases of LPFB and complete (third‐degree) heart block were detected.

#### 3.3.6 Premature Atrial Complexes

PACs were identified in 0.3% (*n* = 21) of participants, with a numerically higher prevalence in females (0.4%) than in males (0.2%), though this difference was not statistically significant (*p* = 0.191) (Table 2 and Figure 2). These findings suggest that supraventricular ectopy is uncommon and similarly distributed between male and female first‐year Vietnamese university students.

#### 3.3.7 Wolff–Parkinson–White Pattern

The WPW pattern was an exceedingly rare ventricular pre‐excitation anomaly in this cohort, identified in only 6 individuals overall (< 0.1%; 95% CI: 0.04%–0.23%). When stratified by sex, the baseline prevalence was numerically higher in males (5 cases, 0.2%; 95% CI: 0.07%–0.47%) than in females (1 case, < 0.1%; 95% CI: 0.00%–0.19%). However, this absolute difference did not reach the threshold for conventional statistical significance (*p* = 0.092).

#### 3.3.8 Prolonged and Short QTc

The prevalence of clinically prolonged QTcB intervals was evaluated using sex‐specific thresholds (≥ 480 ms for females and ≥ 470 ms for males). Under these criteria, the overall prevalence of prolonged QTcB in the freshman cohort was 0.3% (*n* = 19).

Although mean QTcB values were significantly higher in females, the proportion of individuals meeting criteria for clinically prolonged QTcB did not differ significantly between sexes. Prolonged QTcB was identified in 0.3% of female students (*n* = 12) and 0.3% of male students (*n* = 7). Statistical analysis demonstrated no significant association between sex and the presence of prolonged QTcB (*p* = 0.6) (Table 2 and Figure 2).

The prevalence of short QTcB intervals was analyzed using sex‐specific thresholds of < 360 ms for females and < 350 ms for males. In this study population, short QTcB was exceptionally uncommon, with an overall prevalence of 0.2% (*n* = 12).

No significant sex‐based difference was observed in the prevalence of short QTcB (*p* = 0.386). Short QTcB was identified in 0.1% of female students (*n* = 5) and 0.3% of male students (*n* = 7). Although the male cohort demonstrated a slightly higher nominal prevalence, short QTcB remained a rare finding overall and showed no significant association with sex among healthy 18‐year‐old individuals.

#### 3.3.9 Sinus Bradycardia

Sinus bradycardia was evaluated using two thresholds: the conventional clinical definition (< 60 bpm) and a more stringent threshold (< 50 bpm). The overall prevalence of sinus bradycardia (< 60 bpm) was 2.7% (*n* = 163). A highly significant sex difference was observed (*p* < 0.001), with male students demonstrating a substantially higher prevalence than female students (4.5%, *n* = 120 vs. 1.3%, *n* = 43, respectively). Using the stricter threshold of < 50 bpm, sinus bradycardia was exceedingly rare, occurring in only three students overall (< 0.1%). All identified cases were observed in male students (0.1% vs. 0% in females). However, owing to the very small number of cases, this difference did not reach statistical significance (*p* = 0.083), despite a continued trend toward male predominance (Table 2 and Figure 2).

### 3.4 Multivariable Analysis of Sex‐Specific Predictors

Multivariable logistic regression models were constructed to evaluate associations between sex and the prevalence of ERP, RBBB, and SA, with adjustment for resting HR, age, and BMI (Table 3).

**TABLE 3:** Multivariable logistic regression analysis for ERP, RBBB, and SA.

Predictor	ERP adjusted OR (95% CI)	*p*	RBBB adjusted OR (95% CI)	*p*	SA adjusted OR (95% CI)	*p*
Age (per year)	0.84 (0.64–1.08)	0.178	0.85 (0.62–1.14)	0.279	0.64 (0.54–0.76)	< 0.001
Male sex	37.7 (17.2–107)	< 0.001	13.1 (7.55–25.2)	< 0.001	1.01 (0.84–1.21)	0.896
Heart rate	0.95 (0.94–0.96)	< 0.001	1.00 (0.98–1.01)	0.600	0.94 (0.93–0.95)	< 0.001
BMI	0.97 (0.90–1.05)	0.417	0.95 (0.87–1.03)	0.249	1.03 (1.00–1.07)	0.085

Abbreviations: BMI, body mass index; CI, confidence interval; ERP, early repolarization pattern; OR, odds ratio; RBBB, right bundle branch block; SA, sinus arrhythmia.

For ERP, male sex was strongly associated with higher odds of ERP (aOR 37.7, 95% CI 17.2–107; *p* < 0.001). Higher resting HR was independently associated with lower odds of ERP (aOR 0.95 per bpm increase, 95% CI 0.94–0.96; *p* < 0.001). Similarly, male sex was strongly associated with RBBB (aOR 13.1, 95% CI 7.55–25.2; *p* < 0.001), whereas resting HR was not significantly associated with RBBB (aOR 1.00 per bpm increase, 95% CI 0.98–1.01; *p* = 0.600). Neither age nor BMI was significantly associated with ERP or RBBB (Table 3).

For SA, sex was not independently associated with its prevalence (aOR 1.01, 95% CI 0.84–1.21; *p* = 0.896). Higher resting HR was associated with lower odds of SA, with a 6% reduction in odds per 1‐bpm increase (aOR 0.94, 95% CI 0.93–0.95; *p* < 0.001). Older age was also associated with lower odds of SA, corresponding to a 36% reduction in odds per year (aOR 0.64, 95% CI 0.54–0.76; *p* < 0.001). BMI was not significantly associated with SA (aOR 1.03; *p* = 0.085).

Sensitivity analyses using Firth penalized logistic regression (Table S1) yielded estimates broadly consistent with those from conventional maximum‐likelihood models. For ERP, the association between male sex and ERP remained statistically significant (OR 34.46, 95% CI 16.17–92.09), compared with an OR of 37.7 (95% CI 17.2–107) in the conventional model. For RBBB, the corresponding estimates were similarly stable (aOR 12.65, 95% CI 7.33–23.92, vs. aOR 13.1, 95% CI 7.55–25.2). Estimates for age and resting HR were also similar between approaches, while BMI remained non‐significant in both models. Overall, Firth penalisation did not materially alter the principal associations observed in the conventional models.

McFadden's pseudo‐R^2^ values were 0.200 for ERP, 0.108 for RBBB, and 0.105 for SA. The Hosmer–Lemeshow test showed no evidence of poor calibration for the ERP (*p* = 0.789) or RBBB (*p* = 0.658) models. For SA, the Hosmer–Lemeshow test was statistically significant (*p* < 0.001), indicating a discrepancy between observed and predicted probabilities.

Quadratic age terms were not statistically significant for ERP (*p* = 0.508), RBBB (*p* = 0.987), or SA (*p* = 0.795), providing no evidence of non‐linear associations with age. Sex‐by‐age interaction terms were not statistically significant for RBBB (*p* = 0.415) or SA (*p* = 0.089). For ERP, the sex‐by‐age interaction was statistically significant (*p* = 0.028); however, inclusion of the interaction term resulted in quasi‐complete separation and substantial model instability because of the sparse number of ERP events. Therefore, the additive model was retained as the primary model, and the ERP interaction was interpreted cautiously.

## 4 Discussion

This large cross‐sectional study of 6099 healthy first‐year Vietnamese university students demonstrates sex‐related differences across quantitative ECG parameters and qualitative ECG variants.

### 4.1 Core ECG Parameters

All four primary ECG parameters differed significantly between sexes. Females had a higher mean resting HR than males (84.64 vs. 81.55 bpm; *p* < 0.001), consistent with previously reported sex‐related differences in resting sinus node and autonomic function (Li et al. 2025; Carbone et al. 2020). Males had longer PR intervals (141.99 vs. 138.99 ms) and wider QRS complexes (91.44 vs. 80.81 ms; both *p* < 0.001), consistent with previously reported sex differences in atrioventricular and intraventricular conduction (van der Ende et al. 2017; Bessem et al. 2017; Mirahmadizadeh et al. 2021). These differences have also been discussed in relation to sex‐related variation in cardiac ion channel and conduction tissue gene expression (Kurokawa et al. 2016).

Sinus bradycardia (< 60 bpm) was more common in males than females (4.5% vs. 1.3%; *p* < 0.001), corresponding to a 3.5‐fold difference. Using the stricter < 50 bpm threshold, all three identified cases occurred in males, although the difference was not statistically significant (*p* = 0.083) because of the small number of events. The higher prevalence of bradycardia in males despite their lower mean resting HR may reflect differences in the underlying HR distributions, with more males falling below the predefined threshold. This finding is consistent with reported sex‐related differences in autonomic modulation of the sinoatrial node (Huikuri et al. 1996). The observed association between resting HR and SA in this cohort (*p* < 0.001) provides additional evidence of the relevance of resting autonomic state to these ECG phenotypes.

Females had longer Bazett‐corrected QT intervals than males (423.54 vs. 406.46 ms; *p* < 0.001), a difference of approximately 17 ms, consistent with the approximately 18‐ms sex‐related QTcB difference reported at the onset of adulthood by Rautaharju and colleagues (Rautaharju et al. 1992). Despite this difference in mean QTcB, the prevalence of prolonged QTcB did not differ between sexes when sex‐specific thresholds were applied (0.3% in both groups). This finding supports the use of sex‐specific QTcB criteria when interpreting ventricular repolarization in young adults (Patel et al. 2010; Vink et al. 2018). Overall, prolonged QTcB was rare, consistent with previous studies of healthy young populations (Kobza et al. 2009). Short QTcB was even less common (0.2%), with no significant sex difference; although prevalence was slightly higher in males, the small number of cases limits interpretation.

### 4.2 Sinus Arrhythmia

SA was the most prevalent ECG variant identified in this cohort (20.7% overall). In unadjusted analyses, a crude sex difference in SA was observed (22.7% vs. 19.2%; *p* < 0.001); however, this association was attenuated after adjustment for resting HR (aOR = 1.01, 95% CI: 0.84–1.21, *p* = 0.896). Because HR and SA are physiologically related, resting HR may represent part of the pathway underlying the observed association rather than a conventional confounder. As a vagally mediated rhythm, SA reflects the cyclical inhibition and restoration of parasympathetic tone during the respiratory cycle (Soos and McComb 2026; Simoyi and Simoyi 2020). Its high prevalence in this healthy young cohort aligns with their expected autonomic capacity. Consistent with this shared physiological underpinning, lower resting HR was strongly associated with the presence of SA (aOR = 0.94, *p* < 0.001). Because HR and SA are intrinsically linked via cardiac vagal control, adjustment for resting HR allowed assessment of the association between sex and SA conditional on resting HR.

Notably, in unadjusted analyses, a crude sex difference in SA was observed; however, this association was fully attenuated after adjusting for resting HR (aOR = 1.01, *p* = 0.896). This suggests that baseline differences in resting HR likely mediate the apparent sex patterns in SA. This provides important context when contrasting our non‐athlete data with athletic cohorts, where Corîci and colleagues found SA to be more common in female athletes (21.8% vs. 12.6%; *p* = 0.03) (Corîci et al. 2018), suggesting that variations in resting bradycardia or athletic training—rather than isolated biological sex—primarily influence SA prevalence.

Finally, HR variability is known to decline with age. Interestingly, even within our narrow cohort, older age was cross‐sectionally associated with lower odds of SA (aOR = 0.64, *p* < 0.001). A sensitivity analysis restricted strictly to the dominant 17–19‐year age range confirmed this association was not driven by older outliers (aOR = 0.66, *p* < 0.001). However, given our cross‐sectional design, future longitudinal studies are required to determine whether this reflects ongoing autonomic development or cohort‐specific variation.

### 4.3 Early Repolarization Pattern

ERP showed the most pronounced sex disparity in the cohort, present in 6.1% of males and only 0.1% of females (approximately 40‐fold difference; *p* < 0.001). The male predominance is consistent with prior literature: Sun and colleagues reported a 13‐fold higher ERP prevalence in men in a Chinese community population (2.6% vs. 0.2%; *p* < 0.001), with male sex showing the strongest independent association (OR 17.09) (Sun et al. 2017). The higher prevalence observed in our younger cohort (6.1% in males vs. 2.6%) is consistent with evidence of an inverse association between age and ERP prevalence. A large meta‐analysis reported a significant inverse correlation between age and ERP prevalence (Pearson *r* = −0.39; *p* = 0.012), with the greatest difference observed between individuals aged 20–29 years and those aged over 50 years (mean difference 9.7%; *p* = 0.019) (Elenizi et al. 2025). ERP prevalences of 18%–24% have been reported in studies focusing on similarly young populations. Ethnicity also modulates ERP expression. The same meta‐analysis documented a significantly higher mean ERP prevalence in Black populations (22.7% ± 11.7%) versus the general population (8.98% ± 7.6%; *p* < 0.001) (Elenizi et al. 2025). The prevalence observed in our Vietnamese cohort falls within the range reported across these populations, supporting the value of considering population‐specific reference data when interpreting ERP.

In multivariable analysis, male sex remained strongly associated with ERP even after adjustment for resting HR (aOR 37.7, 95% CI 17.2–107; *p* < 0.001). This persistent association suggests that differences in resting HR alone do not account for the observed sex difference. Sex‐related electrophysiological factors, including potential effects of sex hormones on ventricular repolarization, may contribute, although these mechanisms were not directly assessed in the present study.

### 4.4 Bundle Branch Blocks

RBBB was identified in 4.4% of males versus 0.3% of females (*p* < 0.001), with IRBBB accounting for > 90% of all cases in both sexes (4.2% males, 0.3% females). CRBBB was rare but significantly more prevalent in males (0.3% vs. 0.1%; *p* = 0.047). The male predominance in CRBBB and IRBBB replicates findings from the Copenhagen City Heart Study (RBBB 1.4% men vs. 0.5% women; IRBBB 4.7% vs. 2.3%) (Bussink et al. 2013), Finnish health surveys (MacLachlan et al. 2025), Spanish cohort data (Alventosa‐Zaidin et al. 2019), and Pakistani (Nasir et al. 2025) and Indian population studies (Hingorani et al. 2012). The lower absolute RBBB rates in our student cohort compared to middle‐aged European populations suggest the age‐dependence of conduction delays, which accumulate progressively with conduction system fibrosis (Ryan et al. 2026). Male sex also remained independently associated with RBBB after adjustment for resting HR (aOR 13.1, 95% CI 7.55–25.2; *p* < 0.001), reinforcing the contribution of sex‐related structural and conduction system differences beyond autonomic influences alone. No cases of LBBB were identified in this young healthy cohort, consistent with the recognized age‐related increase in LBBB prevalence reported in other populations (Haataja et al. 2013).

### 4.5 PACs, VPCs, and AV Conduction Delays

PACs occurred in 0.3% of participants with no significant sex difference. Similarly VPCs were identified in 0.3% of participants without a significant sex difference and were almost exclusively isolated single ectopic beats. Amir et al. reported a similarly low PVC prevalence of 1.1%, although their study observed a relatively higher prevalence among females (Amir et al. 2019). AV conduction abnormalities were also uncommon occurring in 0.3% of the cohort predominantly as first‐degree AV block with similar prevalence between sexes. These findings are lower than those reported in older community populations; first‐degree AV block has been reported in 0.68%–2% of Chinese cohorts with mean ages of 41–47 years (Shan et al. 2021). The lower prevalence observed in our cohort is consistent with the young healthy asymptomatic characteristics of the study population. LAFB showed a non‐significant trend toward higher prevalence in males (0.2% vs. < 0.1%; *p* = 0.092), consistent with previously reported male predominance of fascicular blocks (Ahmadi et al. 2023). No cases of third‐degree AV block and LPFB were detected in our study population.

### 4.6 WPW Pattern

The overall prevalence of WPW pattern in our cohort was < 0.1%, which is at or below the lower end of the 0.1%–0.3% prevalence range reported in large‐scale screening studies. In the landmark epidemiological study by Guize and colleagues involving 138,048 subjects, a higher prevalence of WPW pattern was reported among males (Guize et al. 1985). In our cohort, WPW pattern was numerically more common in males than females (0.2% vs. < 0.1%; *p* = 0.092), although this difference was not statistically significant. However, only six cases were identified, limiting the precision of sex‐specific prevalence estimates and the ability to assess differences between sexes. The identification of these six cases nevertheless illustrates that pre‐excitation can be detected even in an otherwise healthy young population.

### 4.7 Absence of Brugada Pattern

No Brugada‐type ECG pattern was identified in this cohort. Brugada syndrome is an important cause of sudden cardiac death in South and Southeast Asia (Nademanee et al. 1997). The reported prevalence of Brugada‐type ECG patterns ranges from 0.05% in Japan to 0.19%–0.26% in European healthy populations (Hermida et al. 2000), with a marked male predominance. Gallagher et al. identified Brugada‐type ECG patterns in 0.26% of healthy individuals, exclusively in males (Gallagher et al. 2008). The absence of Brugada‐type patterns in our cohort may reflect the low prevalence of the phenotype and the intermittent nature of ECG manifestations, as well as the use of a single resting ECG without pharmacological provocation.

### 4.8 Absence of Atrial Fibrillation

No cases of atrial fibrillation were detected, consistent with the strong age‐related increase in AF prevalence. Reported prevalence rises from approximately 1%–4% at age 60 to 6%–15% at age 80 (Andrade et al. 2018), and male sex confers a 1.5‐fold higher incidence (Framingham: 3.8 vs. 1.6 cases per 1000 person‐years in men vs. women) (Feinberg et al. 1995). Given the young age and absence of structural heart disease in our cohort, the absence of AF was therefore not unexpected.

### 4.9 Clinical Implications and Limitations

The consistent sex‐related differences observed across quantitative ECG parameters and qualitative ECG variants in this study may be relevant to the interpretation of ECG findings in healthy young Vietnamese university students. Differences between our findings and previously reported ECG characteristics in other populations suggest that reference ranges derived from different demographic groups may not fully capture the ECG characteristics of this population. The higher prevalence of ERP, IRBBB, and SA among male students, together with the longer QTc observed among female students, highlights the potential value of considering sex when interpreting ECG findings in young Vietnamese university students. These findings may provide a useful reference framework for university‐level and population‐based cardiovascular screening programmes in Vietnam and neighboring countries.

However, this study has several limitations that should be considered when interpreting the findings. First, enrolment was restricted to first‐year university students aged 17–29, limiting generalisability to older adults, community‐based, or non‐academic populations. Second, lifestyle factors known to influence autonomic tone and ECG parameters—including physical activity level, diet, alcohol and tobacco use, sleep quality, and psychological stress—were not systematically assessed. Third, physical fitness was not formally quantified; training status independently modulates HR and QT interval and may confound sex comparisons. Fourth, while the use of hormonal agents was an exclusion criterion, endogenous sex hormone levels were not measured, precluding direct assessment of their influence on repolarisation. Fifth, given the mixed and evolving evidence regarding the influence of the menstrual cycle on ECG parameters, the exclusion of 279 menstruating women may have introduced selection bias rather than fully eliminating a clinically meaningful source of variation. Sixth, the Brugada pattern is known to be intermittent and concealed in a substantial proportion of cases; a single unprovoked resting ECG without pharmacological provocation cannot exclude its presence. Finally, the cross‐sectional design precludes evaluation of temporal changes in ECG parameters or determination of the long‐term prognostic significance of identified ECG variants.

## Author Contributions

T.N.N. contributed to conceptualization, study design, and manuscript drafting. Q.N.M., T.V.T., and S.H.T. contributed to methodology, data analysis, and manuscript revision. A.B.T.T., C.D.C., V.N.H., and B.T.V. contributed to data collection, curation, and interpretation. C.N.T. and A.D.T. contributed to data interpretation and critical revision. All authors reviewed and approved the final manuscript and agreed to be accountable for all aspects of the work, in accordance with ICMJE authorship criteria.

## Funding

The authors have nothing to report.

## Ethics Statement

Patients and the public were not involved in the design, conduct, reporting, or dissemination of this research. The study was approved by the Institutional Review Board of Phenikaa University Hospital, approval/reference number PUH‐IRB‐2025‐082. As the ECG data were collected as part of a mandatory university health‐screening programme and subsequently anonymised for retrospective research use, individual informed consent was waived by the approving committee.

## Conflicts of Interest

The authors declare no conflicts of interest.

## Supporting information


**Table S1:** Sensitivity Analysis Comparing Conventional and Firth‐Penalized Multivariable Logistic Regression Models for Early Repolarization Pattern (ERP) and Right Bundle Branch Block (RBBB). Multivariable logistic regression estimates evaluating independent associations for early repolarization pattern (ERP) and right bundle branch block (RBBB), contrasting conventional maximum‐likelihood estimation with Firth penalized logistic regression. Firth's penalized likelihood approach was conducted as a sensitivity analysis to control for potential small‐sample bias and sparse‐data event numbers (quasi‐complete separation). All models simultaneously adjust for age (per year increase), male sex, resting heart rate (per bpm increase), and body mass index (BMI, per kg/m^2^ increase). Results are presented as adjusted odds ratios (aOR) with corresponding 95% confidence intervals (95% CI) and two‐sided *p*‐values.

## Data Availability

The data that support the findings of this study are available from the corresponding author upon reasonable request.
