# Supplementary material for: Sex‐Specific Prevalence and Distribution of Electrocardiographic Variants in Healthy Vietnamese Freshmen
Source: Ann Noninvasive Electrocardiol. 2026 Oct 22;31(6):e70247. doi: 10.1111/anec.70247 (PMC13688244; doi:10.1111/anec.70247)
Supplement: Supplementary file 1 [file ANEC-31-e70247-s001.docx]

| **Predictor** | **ERP: Firth OR (95% CI)** | **p-value** | **RBBB: Firth OR (95% CI)** | **p-value** | **ERP: Conventional OR (95% CI)** | **p-value** | **RBBB: Conventional OR (95% CI)** | **p-value** |
| --- | --- | --- | --- | --- | --- | --- | --- | --- |
| Age (per year) | 0.841 (0.640–1.070) | 0.169 | 0.866 (0.633–1.145) | 0.335 | 0.84 (0.64–1.08) | 0.178 | 0.85 (0.62–1.14) | 0.279 |
| Male sex | 34.455 (16.173–92.090) | <0.001 | 12.652 (7.331–23.922) | <0.001 | 37.7 (17.2–107) | <0.001 | 13.1 (7.55–25.2) | <0.001 |
| Heart rate | 0.951 (0.938–0.963) | <0.001 | 0.997 (0.985–1.009) | 0.603 | 0.95 (0.94–0.96) | <0.001 | 1.00 (0.98–1.01) | 0.600 |
| BMI | 1.069 (0.988–1.156) | 0.096 | 0.937 (0.859–1.022) | 0.141 | 0.97 (0.90–1.05) | 0.417 | 0.95 (0.87–1.03) | 0.249 |

***Supplementary Table 1: Sensitivity Analysis Comparing Conventional and Firth-Penalized Multivariable Logistic Regression Models for Early Repolarization Pattern (ERP) and Right Bundle Branch Block (RBBB)****. Multivariable logistic regression estimates evaluating independent associations for early repolarization pattern (ERP) and right bundle branch block (RBBB), contrasting conventional maximum-likelihood estimation with Firth penalized logistic regression. Firth’s penalized likelihood approach was conducted as a sensitivity analysis to control for potential small-sample bias and sparse-data event numbers (quasi-complete separation). All models simultaneously adjust for age (per year increase), male sex, resting heart rate (per bpm increase), and body mass index (BMI, per kg/m² increase). Results are presented as adjusted odds ratios (aOR) with corresponding 95% confidence intervals (95% CI) and two-sided p-values.*
